# Signal Transducer and Activator of Transcription–3 Induces MicroRNA-155 Expression in Chronic Lymphocytic Leukemia

**DOI:** 10.1371/journal.pone.0064678

**Published:** 2013-06-04

**Authors:** Ping Li, Srdana Grgurevic, Zhiming Liu, David Harris, Uri Rozovski, George A. Calin, Michael J. Keating, Zeev Estrov

**Affiliations:** 1 Department of Leukemia, The University of Texas MD Anderson Cancer Center, Houston, Texas, United States of America; 2 Department of Experimental Therapeutics, The University of Texas MD Anderson Cancer Center, Houston, Texas, United States of America; University of Manitoba, Canada

## Abstract

MicroRNA (miR) abnormalities play a key role in the pathogenesis of chronic lymphocytic leukemia (CLL). High levels of miR-155 have been detected in human neoplasms, and overexpression of miR-155 has been found to induce lymphoma in mice. High levels of miR-155 were detected in CLL cells and STAT3, which is known to induce miR-21 and miR-181b-1 expression, is constitutively activated in CLL. Given these findings, we hypothesized that STAT3 induces miR-155. Sequence analysis revealed that the miR-155 promoter harbors two putative STAT3 binding sites. Therefore, truncated miR-155 promoter constructs and STAT3 small interfering RNA (siRNA) were co-transfected into MM1 cells. Of the two putative binding sites, STAT3-siRNA reduced the luciferase activity of the construct containing the 700–709 bp STAT3 binding site, suggesting that this site is involved in STAT3-induced transcription. Electrophoretic mobility shift assay confirmed that STAT3 bound to the miR-155 promoter in CLL cells, and chromatin immunoprecipitation and luciferase assay confirmed that STAT3 bound to the 700–709 bp but not the 615–624 bp putative STAT3 binding site in CLL cells. Finally, STAT3-small hairpin RNA downregulated miR-155 gene expression, suggesting that constitutively activated STAT3 binds to the miR-155 gene promoter. Together, these results suggest that STAT3 activates miR-155 in CLL cells.

## Introduction

B-cell chronic lymphocytic leukemia (CLL), which is characterized by a progressive accumulation of leukemia cells that co-express CD5 and CD19 surface antigens [1], is the most common hematologic malignancy in the Western hemisphere. Despite significant progress in CLL research and novel therapies for the disease, CLL remains incurable, and its pathobiology is still not fully understood [2].

MicroRNAs (miRNAs; miRs) are small noncoding RNAs, 19–24 nucleotides in length, that regulate gene expression. MiRs are expressed aberrantly in human neoplasms including leukemia and lymphoma. Aberrantly expressed miRs repress multiple genes by inhibiting translation, cleaving mRNA, and guiding deadenylation that initiates mRNA decay [3]. Approximately 1000 human miRs regulate more than 30% of the protein-coding genes at the posttranscriptional and translational levels, and several miRs regulate multiple cellular processes, thereby playing an important role in cell and tissue homeostasis [4].

In CLL, miRs function as oncogenes or tumor suppressors [5], [6]. The loss of the miRNAs miR-15a and miR-16-1 in patients with the 13q deletion contributes to the pathogenesis of the disease [7], [8], and altered miR expression is associated with disease progression and poor prognosis [9]. miRs are also involved in normal B-cell activation [10]. Activated B cells and CLL cells exhibit similar miR expression profiles that include the upregulation of miR-34a, miR-155, and miR-342-3p and the downregulation of miR-103, miR-181a, and miR-181b [10]. MiR-155 has been found to play a role in autoimmunity and tumorigenesis [11], and its overexpression induced lymphoma in mice [12]. However the mechanism underlying miR-155 expression in CLL cells is unknown.

In CLL, as in other neoplasms, miRs activate inflammatory pathways. MiR-21 and miR-29a bind as ligands to receptors of the Toll-like receptor family members [13]. Remarkably, miR-21 transcription is activated by signal transducer and activator of transcription-3 (STAT3) [14], which is known to contribute to the pathogenesis of CLL [15]. Constitutive STAT3 phosphorylation is required for the survival and proliferation of a number of tumor cells. In CLL, STAT3 is constitutively phosphorylated on serine 727 residues [15], [16], and similar to phosphotyrosine STAT3, phosphoserine STAT3 shuttles to the nucleus, binds to DNA, and activates the transcription of STAT3 target genes [15]. We hypothesized that, because miR-155 is overexpressed in CLL [9], [17], [18], [19] and STAT3 is associated with the induction of several miRs in various cell types [14], [20], STAT3 induces the expression of miR-155 in CLL cells.

## Materials and Methods

### B-cell CLL Cell Fractionation

Peripheral blood (PB) cells from patients who were treated at The University of Texas MD Anderson Cancer Center Leukemia Clinic were processed after Institutional Review Board approval and a written patient informed consent were obtained. To isolate low-density cells, PB cells were fractionated using Ficoll Hypaque 1077 (Sigma-Aldrich, St. Louis, MO). More than 90% of the PB lymphocytes obtained from these patients were CD19+/CD5+ as assessed by flow cytometry.

### Cell Culture

Fractionated CLL cells were maintained in DMEM (Sigma-Aldrich) supplemented with 10% FBS (Hyclone, Logan, UT). For some experiments CLL cells were incubated with recombinant human (rh) interleukin (IL)-6 (BioSource International, Camarillo, CA). Human multiple myeloma MM1 cells (American Type Culture Collection, Rockville, MD) were maintained in RPMI 1640 (Sigma-Aldrich) supplemented with 10% fetal bovine serum in a humidified, 5% CO_2_ atmosphere at 37°C.

### Generation of Luciferase Reporter Plasmids

The human miR-155 promoter was generated using polymerase chain reaction (PCR). Genomic DNA isolated from human PB mononuclear cells was used as a template. The PCR products were purified using the QIAquick Gel Extraction Kit (Qiagen, Inc., Valencia, CA). Human miR-155 promoter PCR primers were designed according to the sequence of the miR-155 5′-flanking region (Ensembl Resource, www.ensembl.org). The miR-155 promoter PCR primers included a forward primer starting at bp −728 GCG GCG GGT ACC CAA AGC CAC TGG GTA GTG CTT, a reverse primer starting at bp −1 GCG GCG AGA TCT CAT ACA GCC TAC AGC AAG CCT, and two truncated forward primers located at −653 GCG GCG GGT ACC GTA GGC AAA CCT CCA TTG CTT and −574 GCG GCG GGT ACC AAT TCA GCC TGA ACC CTA CCC. The amplified fragments were cloned to the *BglII-* and *KpnI-*digestion sites of the pGL4.17 vector (Promega), which contains a luciferase reporter gene. The sequences of all constructs were verified by an automated sequencing service (Seqwright, Houston, TX).

### Transfection of Cells and Luciferase Assay

To determine to which of the two putative STAT3 binding sites STAT3 binds, MM1 cells were transfected by electroporation as described previously [21]. Before electroporation, 5 µl of siPORT NeoFX transfection reagent (Ambion) and 30 nmol STAT3–small interfering RNA (siRNA) or scrambled siRNA (Applied Biosystems) diluted in 100 µl Opti-MEM medium (Invitrogen) were incubated at room temperature for 10 min. MM1 cells (5×10^6^) suspended in 0.2 ml of Opti-MEM medium containing the STAT3 siRNA and siPORT NeoFX transfection reagent were incubated at room temperature for 1 hour. Following incubation, 2 µg of each of the specific reporter constructs was added. Electroporation was then conducted using the Gene Pulser Xcell Electroporation System (Bio-Rad, Hercules, CA). Following electroporation, the co-transfected cells were maintained in RPMI 1640 supplemented with 10% fetal calf serum in a humidified, 5% CO_2_ atmosphere at 37°C. As control, the luciferase activity of untreated and IL-6-treated MM1 cells was assessed, as previously described [21].

Luciferase activity was assessed 48 hours after transfection using the Dual-Luciferase Reporter Assay System (Promega) and a SIRIUS luminometer V3.1 (Berthold Detection Systems, Pforzheim, Germany). The luciferase activity of each of the human miR-155 promoter constructs was determined by calculating the constructs’ luciferase activity relative to the activity of the Renilla luciferase produced by the pRL-SV40 vector, which was used as a control. At least three separate experiments were conducted, and the means ± standard deviations of the luciferase activities were calculated.

### Chromatin Immunoprecipitation Assay

Chromatin immunoprecipitation (ChIP) was performed to determine which putative binding site binds STAT3. The ChIP assay was performed using the SimpleChIP Enzymatic Chromatin IP Kit (Cell Signaling Technology) according to the manufacturer’s instructions. Briefly, cells were cross-linked with 1% formaldehyde for 10 minutes at room temperature, harvested, and then incubated on ice for 10 minutes in lysis buffer. Nuclei were pelleted and digested by micrococcal nuclease. Following sonication and centrifugation, sheared chromatin was incubated with anti-STAT3 or rabbit serum (negative control) overnight at 4°C. Then, protein G beads were added, and the chromatin was incubated for 2 hours. An aliquot of chromatin that was not incubated with an antibody was used as the input control sample.

Antibody-bound protein/DNA complexes were eluted and subjected to PCR analysis. The primers used to amplify the miR-155 promoter’s putative STAT3 binding sites are depicted in Figure S1A. The primers to amplify the human STAT3 promoter were F: 5′-CCG AAC GAG CTG GCC TTT CAT-3 and R: 5′-GGA TTG GCT GAA GGG GCT GTA-3, which generated an 86-bp product; primers to amplify the p21/WAF1 promoter were F: 5′-TTG TGC CAC TGC TGA CTT TGTC-3 and R: 5′-CCT CAC ATC CTC CTT CTT CAG GCT-3, which generated a 303-bp product; primers to amplify the c-Myc promoter were F: 5′-TGA GTA TAA AAG CCG GTT TTC-3 and R: 5′-AGT AAT TCC AGC GAG AGG CAG-3, which generated a 63-bp product, and to amplify the VEGF promoter: R: 5′-1453 CCT GGA AAT AGC CAG GTC AG F: 3–1272 CTT CTC CAG GCT CAC AGC TT. The human RPL30 gene primers were provided by Cell Signaling Technology.

### Electrophoretic Mobility Shift Assay

Electrophoretic mobility shift assay (EMSA) was performed to validate binding of STAT3 to the miR-155 promoter. Non-denatured cellular nuclear extracts were prepared using the NE-PER extraction kit (Thermo Scientific, Pierce). Two micrograms of nuclear protein extracts were incubated with biotin-labeled ROR1 promoter DNA probes in binding buffer for 30 minutes on ice. All probes were synthesized by Sigma Genosys (The Woodlands, TX). The sequences of the probes used are depicted in Figure S1B. Following incubation, the samples were separated on a 5% polyacrylamide gel in Tris-borate ethylenediaminetetraacetic acid, transferred to a nylon membrane, and fixed by ultraviolet cross-linking. The biotin-labeled probe was detected with streptavidin-horseradish peroxidase (Gel–Shift Kit; Panomics, Fremont, CA). The competition control consisted of up to a 7-fold excess of cold, unlabeled probe combined with biotin-labeled probes.

To determine the effect of antibodies on protein-DNA binding, we incubated 1 µg of monoclonal mouse anti-human STAT3 (BD Biosciences; Cell Signaling Technology) or mouse anti-human phosphoserine STAT3 (Cell Signaling Technology) antibody with the nuclear extracts for 30 minutes on ice and then added the biotin-labeled DNA probe. The isotypic control in both experiments was mouse immunoglobulin G1 (IgG)(BD Biosciences) [15], [22].

### RNA Purification and qRT-PCR

RNA was isolated using an RNeasy purification procedure (Qiagen). RNA quality and concentration were analyzed using a NanoDrop 1000 spectrophotometer (Wilmington, Delaware). Total RNA (500 ng) was subjected to one-step quantitative reverse transcriptase PCR (qRT-PCR) using the ABI Prism 7700 sequence detection system (Applied Biosystems, Foster City, CA) with TaqMan gene expression assays for Bcl-2, c-Myc, cyclin D1, p21/WAF1, vascular endothelial growth factor (VEGF)-C, miR-155, and S18 according to the manufacturer’s instructions. Samples were run in triplicate, and relative quantification was performed using the comparative C_T_ method as described previously [21].

### STAT3 siRNA Transfection

Knockdown of endogenous STAT3 was performed as described previously [21]. Pre-designed siRNA and scrambled siRNA were obtained from Ambion Applied Biosystems. The siRNA sequences used to target exons 14 and 15 of the human STAT3 gene were antisense 5′-GGG AAG CAU CAC AAU UGG CTC-3′ and sense 5′-GCC AAU UGU GAU GCU UCC CTT-3′. siRNA (50 nM) was mixed with siPORT NeoFX Transfection Agent (Ambion) and transfected by electroporation into CLL cells. The transfected CLL cells were then co-cultured with mesenchymal stroma cells. qRT-PCR was performed 72 hours after transfection.

## Results

### *MiR-155* Promoter Harbors Putative STAT3 Binding Sites

**Figure 1:**
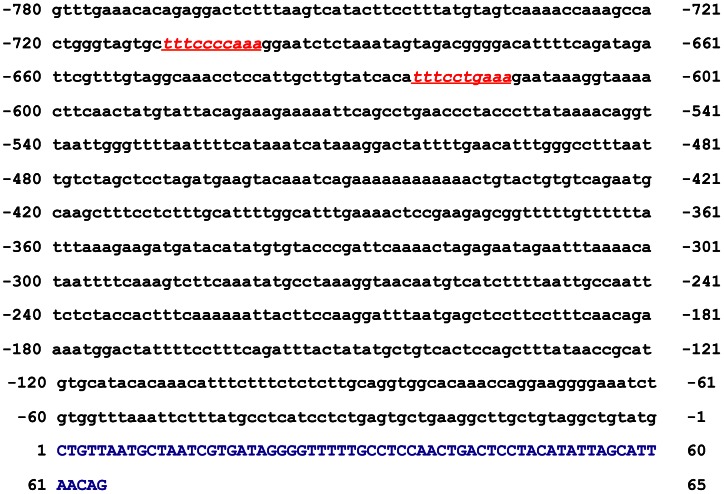
Sequence of the miR-155 gene promoter. Sequence analysis of the miR-155 promoter detected two putative STAT3 binding sites (red).

We first sought to determine whether miR-155 has any STAT3 binding sites. Typically, phosphorylated STAT3 binds to the γ-interferon activation sequence (GAS)-like element, also referred to as the sis-inducible element, in the promoters of various genes [23]. Because STAT3 is constitutively activated in CLL and activates several STAT3 target genes [15] and miR-155 is overexpressed in CLL [19], we sought to determine whether miR-155 has any STAT3 binding sites. Sequence analysis identified two GAS-like elements in the miR-155 promoter (Fig. 1), suggesting that STAT3 binds to the miR-155 promoter in CLL cells.

### Transfection of the −729 bp miR-155 Gene Promoter Fragment Induces Luciferase Activity

**Figure 2:**
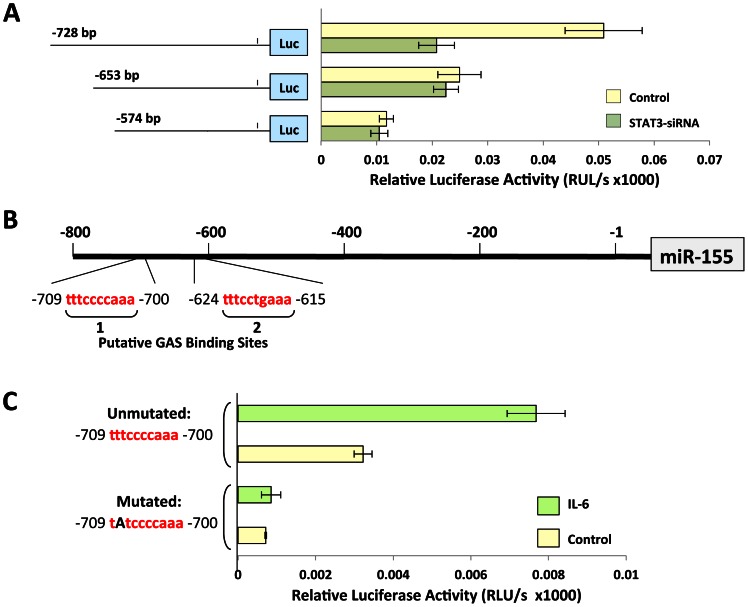
Luciferase activity of MM1 cells transfected with miR-155 promoter fragments. **A,** MM1 cells were co-transfected with miR-155 promoter fragments and either STAT3-siRNA or scrambled siRNA (control). Luciferase activity was assessed after 48 hours. STAT3-siRNA markedly reduced the luciferase activity of the −728 bp fragment but not the shorter −653 and −574 bp fragments. Means ± standard deviations of relative luciferase activity units from at least 3 different experiments are shown. **B,** Schematic diagram of the reporter gene of the human mir-155 promoter. Fragment −728 bp contains the putative STAT3 binding sites 1 and 2, fragment −635 bp contains the putative STAT3 binding site 2, and fragment −574 bp does not contain a STAT3 binding site. **C,** MM1 cells were transfected with the unmutated or the t-to-a mutated −728 bp fragment. Incubation of MM1 cells with 50 ng/ml IL-6 significantly enhanced the luciferase activity of MM1 cells transfected with the unmutated but not with the mutated −728 bp fragment. As shown, the luciferase activity of IL-6-stimulated cells transfected with the unmutated −728 bp fragment was significantly higher (P<0.0006) than that of MM1 cells transfected with the mutated fragment. Means ± standard deviations of relative luciferase activity units from 3 different experiments are depicted.

Because low levels of activated STAT3 were detected in MM1 cells [21] and luciferase activity was high in MM1 cells transfected with miR-155 promoter transcripts, we co-transfected MM1 cells with miR-155 promoter fragments of different lengths with either STAT3-siRNA or scrambled siRNA (control) and assessed the relative luciferase activity 48 hours after transfection. Luciferase activity analysis revealed that co-transfection with STAT3-siRNA downregulated the luciferase activity of the −728 bp fragment but not the −653 bp or −574 bp fragments (Fig. 2A). The −653 bp fragment contained the putative STAT3 binding site 2, but the −574 bp fragment does not contain any STAT3 binding site (Fig. 2B), which suggests that the STAT3 binding site 1 drives STAT3-induced luciferase activity in MM1 cells. To further delineate these findings, we assessed the luciferase activity of untreated and IL-6-treated MM1 cells 48 hours after transfection with the −728 bp fragment (Fig. 2C). IL-6, known to induce phosphorylation of STAT3 [21], significantly enhanced the luciferase activity of MM1 cells transfected with the −728 bp fragment but not the luciferase activity of cells transfected with a mutated miR-155 −728 bp fragment (Fig. 2C), suggesting that the luciferase activity of site 1 is specific and driven by STAT3.

### STAT3 Binds to the miR-155 Promoter in CLL Cells

**Figure 3:**
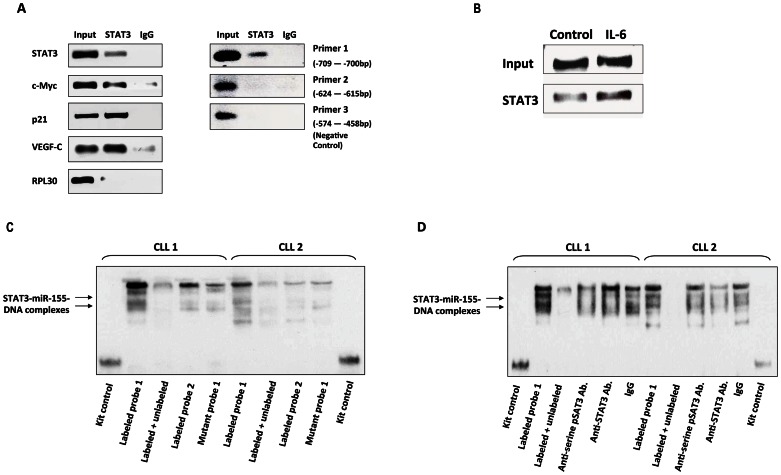
STAT3 binds to the miR-155 promoter. **A,** Left panel: ChIP of CLL cell nuclear protein revealed that STAT3 co-immunoprecipitated with the DNA of the STAT3 target genes STAT3, c-Myc, p21/WAF1, and VEGF-C, but not that of the control gene RPL30. Right panel: Anti-STAT3 antibodies co-immunoprecipitated with the DNA of probe 1 but not that of probes 2 or 3, suggesting that STAT3 binds to the putative STAT3 binding site 1. In both experiments, immunoglobulin G1 (IgG) was used as an isotypic control. **B,** ChIP of CLL cells incubated without or with 30 ng/ml rhIL-6. Using primer 1, we found that the amount of STAT3 DNA that was co-immunoprecipitated with anti-STAT3 antibodies from CLL cells incubated with IL-6 was higher than that from control cells, suggesting that IL-6 enhances the binding of STAT3 to the miR-155 promoter. **C.** EMSA performed using the cells of two CLL patients demonstrated that CLL cell nuclear protein binds to miR-155– biotinylated (labeled) DNA probe 1 and that excess cold, unlabeled probe attenuates this binding. No significant binding was obtained with labeled probe 2. **D,** EMSA performed using the cells of the same two CLL patients demonstrated that CLL cell nuclear protein binds to miR-155–labeled DNA probe 1 and that excess cold, unlabeled probe attenuates this binding. The addition of anti-phosphoserine–STAT3 and anti-STAT3 antibodies revealed a similar effect, suggesting that STAT3 binds the miR-155 promoter at binding site 1.

Because the luciferase activity analysis suggested that STAT3 binds to site 1 in MM1 cells, we sought to determine whether STAT3 binds to site 1 of the miR-155 promoter in CLL cells. ChIP revealed that anti-STAT3 antibodies co-immunoprecipitated the DNA of the STAT3-regulated genes STAT3, c-Myc, VEGF-C, and p21/WAF1 but not the DNA of the control gene RPL30 (Figure 3A, left panel). Anti-STAT3 antibodies also co-immunoprecipitated the DNA of primer 1, which harbored the STAT3 binding site 1, but not the DNA of primer 2, which harbored the STAT3 binding site 2, or the DNA of primer 3, the negative control (Fig. 3A, right panel), suggesting that STAT3 binds to site 1 of the miR-155 promoter.

Because IL-6 activates STAT3 [15], we performed ChIP of CLL cells incubated without or with 30 ng/ml rhIL-6 to determine which putative miR-155 promoter binds STAT3. We found that incubation with IL-6 enhanced the co-immunoprecipitation of STAT3 with anti-STAT3 antibodies, suggesting that IL-6 enhances the binding of STAT3 to the miR-155 promoter (Fig. 3B).

To validate the above findings, we performed EMSA, which revealed that CLL cell nuclear extracts from two different CLL patients bound the labeled probe 1, which harbored the putative STAT3 binding site 1. The addition of excess cold, unlabeled probe significantly attenuated the binding, thus confirming binding specificity (Fig. 3C). In contrast, no significant binding to the labeled probe 2, which harbored the putative STAT3 binding site 2, or the mutated probe 1 was detected. Furthermore, both anti-phosphoserine STAT3 and STAT3 antibodies attenuated the binding, suggesting that STAT3 binds to site 1 of the miR-155 promoter (Fig. 3D).

### STAT3 Activates the miR-155 Promoter in CLL Cells

**Figure 4:**
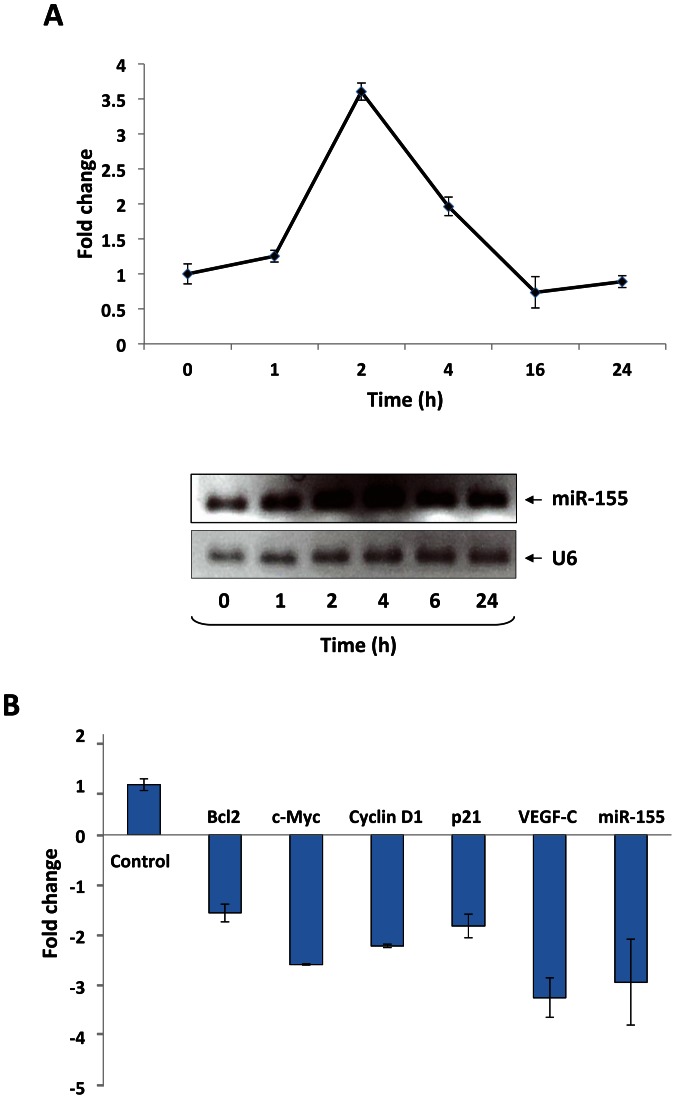
STAT3 activates miR-155 in CLL cells. A, CLL cells were incubated for 20 minutes with 30 ng/ml rhIL-6. Following incubation, cells were collected at different times, and total RNAs were prepared. qRT-PCR (upper panel) and RT-PCR (lower panel) revealed that IL-6 upregulated miR-155 expression levels in a time-dependent manner. The U6 gene was used as an internal control for miRNA expression. **B,** Transfecting CLL cells with STAT3-siRNA at a transfection efficiency of 35% downregulated the expressions of miR-155 and the STAT3-regulated genes Bcl-2, c-Myc, Cyclin D1, p21/WAF1, and VEGF-C.

We next sought to determine whether STAT3 activates miR-155 in CLL cells. Because IL-6 has been found to increase the levels of serine pSTAT3 and induce the phosphorylation of STAT3 on tyrosine residues [15], we incubated CLL cells with 30 mg/ml rhIL-6 for 20 minutes and analyzed their miR-155 expression levels at different times following incubation using RT-PCR and relative qRT-PCR. Both experiments revealed that the miR-155 expression levels 2 hours after incubation were almost 4-fold higher than those at baseline and that miR expression returned to baseline levels at 16 hours, suggesting that IL-6 activates miR-155 expression (Fig. 4A). We then transfected CLL cells with STAT3-siRNA and used relative qRT-PCR to quantitate STAT3-regulated gene mRNA levels. We found that STAT3-shRNA markedly downregulated the expression levels of miR-155 as well as those of the STAT3-regulated genes Bcl-2, c-Myc, cyclin D1, p21/WAF1, and VEGF-C (Fig. 4B).

## Discussion

STAT3 is constitutively activated in CLL cells, and downregulation of STAT3 induces apoptosis of CLL cells [15]. Phosphorylated STAT3 typically binds to GAS-like elements located in the promoter region of various genes [23]. In the present study, we identified two GAS-like elements in the promoter region of miR-155, suggesting that STAT3 binds to the promoter of miR-155. We also identified a 700–709 bp STAT3 binding site in the miR-155 promoter region and found that STAT3 binds to that site in CLL cells. Finally, we found that IL-6, which is known to activate STAT3 in CLL cells [15], upregulated miR-155 expression and that STAT3-siRNA downregulated miR-155 expression. Together, these findings suggest that STAT3 activates miR-155 in CLL.

MiRs modulate developmental processes, cell fate decisions, and homeostasis in several tissues [3]. MiR-155 regulates hematopoietic cell development. Several studies including gene-targeting experiments in mice have elucidated the function of miR-155 in immune B- and T-cell response, cytokine and antibody production, and antigen presentation [24], [25]. The deregulation of endogenous miR-155 function has been implicated in the pathogenesis of human cancers and autoimmune diseases including rheumatoid arthritis, multiple sclerosis, and systemic lupus erythematosus [26]. In murine models, miR-155 overexpression stimulated B-cell proliferation and was associated with lymphoma development [12]. In humans, upregulated miR-155 expression levels have been found in CLL [9], [17], [18] and Hodgkin and non-Hodgkin lymphomas [25], [27], [28], [29].

Mechanisms that alter MiR expression include structural genetic alterations, mutations, single nucleotide polymorphisms, Drosha and Dicer activity, methyltransferases, histone deacetylases, or other miRs [30]. Putative target genes for individual miRs, identified by bioinformatic computational programs, have been validated experimentally [18]. However, information on miR regulation at the transcriptional and posttranscriptional levels is limited. Vargova et al. recently reported that overexpression of v-myb myeloblastosis viral oncogene homolog (MYB) associates with and activates the transcription of the miR-155 promoter in a subset of CLL patients; however, the correlation between MYB and miR-155 expression levels was not strong [19]. Several investigators have reported that STAT3 is associated with the induction of miRs such as miR-21 and miR-181b-1 [14], [20], and our own analysis in a previous study suggested that STAT3 directly regulates the transcription of a number of miRs including miR-155 (Rozovski et al., submitted). Indeed, in the present study, sequence analysis identified two putative STAT3 binding sites in the promoter of the miR-155 gene.

All STAT family members play a key role in organ development and cell proliferation and survival. However, STAT3 is the only STAT family member whose deletion results in embryonic lethality [31]. STAT3 is constitutively activated in CLL cells, and downregulation of STAT3 induces apoptosis of CLL cells [15].

Phosphorylated STAT3 typically binds to GAS-like elements located in the promoter region of various genes [23]. In the present study, we identified two GAS-like elements in the promoter of miR-155, which suggested that STAT3 binds to the promoter of miR-155. Therefore, using MM1 cells, we cloned the promoter of miR-155 and investigated whether STAT3 induces miR-155 expression in CLL cells. By transfecting miR-155 promoter fragments into MM1 cells and assessing their luciferase activity, we identified a STAT3 binding site in the miR-155 promoter. Then, using ChIP and EMSA, we found that STAT3 binds to that 700–709 bp site in the miR-155 promoter in CLL cells. Because IL-6, which is known to activate STAT3 in CLL cells [15], upregulated miR-155 levels, and STAT3-siRNA downregulated the expression miR-155, we concluded that STAT3 activates miR-155.

STAT3 activates pro-inflammatory pathways and plays a crucial role in modulating the epigenetic switch that links inflammation to cancer [14]. STAT3’s activation of miR-155 likely triggers the phosphatidylinositol 3-kinase–protein kinase B pathway, which is known to be activated by miR-155 [32]. Indeed, protein kinase B is activated in CLL cells from most patients [33], and high levels of miR-155 have been found to be associated with high zeta-chain-associated protein kinase 70 expression, a predictor of disease progression [34], and a short duration to treatment initiation [9]. Given the key role miR-155 plays in the pathobiology of CLL, the findings of the present study further highlight the need to develop STAT3 inhibitors as therapeutic agents for CLL.

## Supporting Information

Figure S1 **A,** Primers used to identify the putative STAT3 binding sites and their location in the miR-155 promoter. **B,** Wild-type and mutated probes of the putative GAS binding site 1 and wild-type probe of the putative GAS binding site 2.
(TIF)
