# Supplementary figures and images for: Signal Transducer and Activator of Transcription–3 Induces MicroRNA-155 Expression in Chronic Lymphocytic Leukemia
Source: PLoS One. 2013 Jun 4;8(6):e64678. doi: 10.1371/journal.pone.0064678 (PMC3672147; doi:10.1371/journal.pone.0064678)

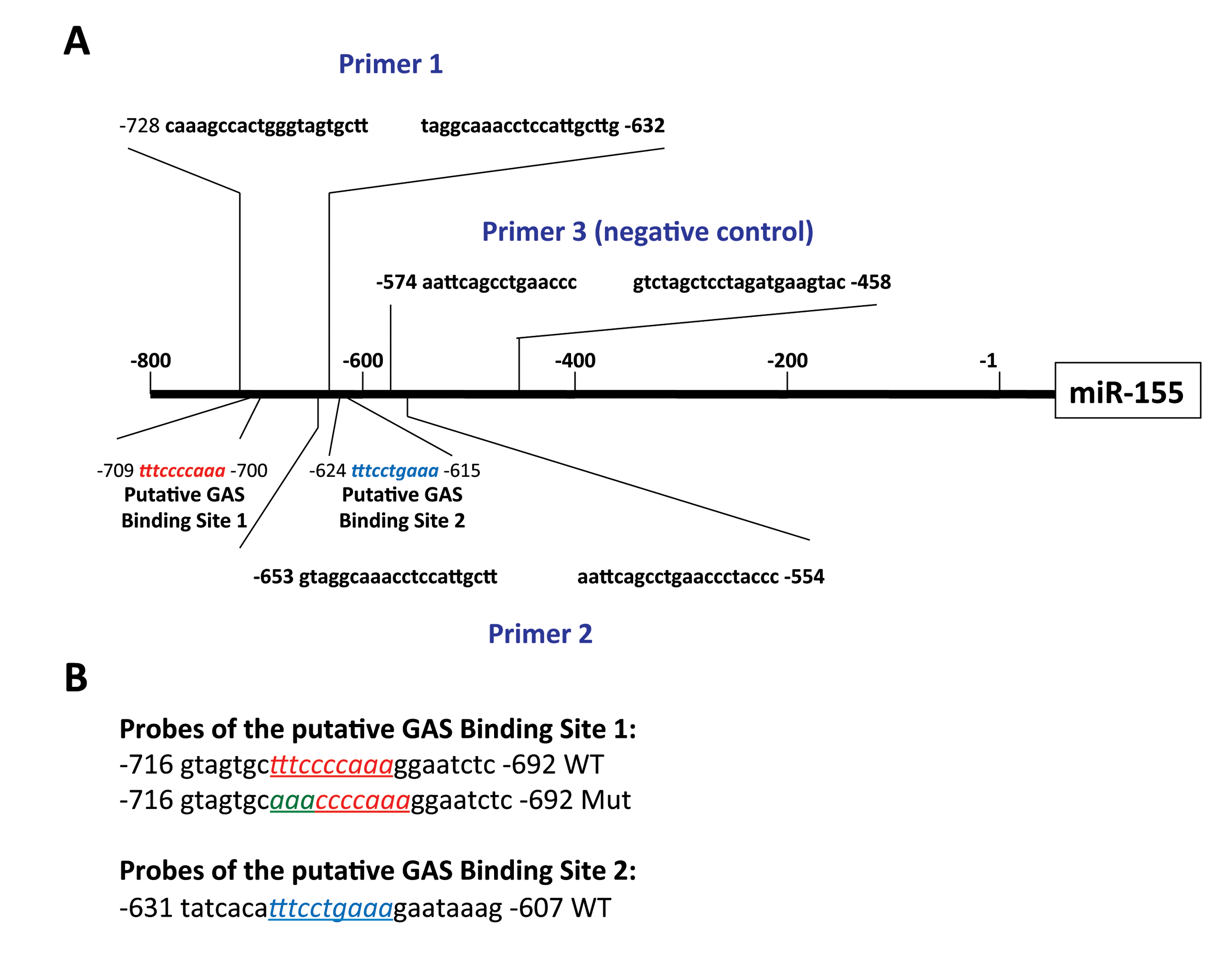

Supplement: Figure S1 — A, Primers used to identify the putative STAT3 binding sites and their location in the miR-155 promoter. B, Wild-type and mutated probes of the putative GAS binding site 1 and wild-type probe of the putative GAS binding site 2. (TIF) [file pone.0064678.s001.tif]
